# The role of shoulder arthroplasty after chronic brucellosis of glenohumeral joint septic arthritis. A case report and literature reviews

**DOI:** 10.1016/j.ijscr.2022.107467

**Published:** 2022-07-30

**Authors:** Bancha Chernchujit, Surasak Srimongkolpitak, Jutatip Kintarak, Yodsawee Pornmeechai

**Affiliations:** a Department of Orthopedics, Faculty of Medicine, Thammasat University, Phaholyothin Frontage Rd, Khlong Nueng, Khlong Luang District, Pathum Thani 12120, Thailand; b Department of Orthopedic, Faculty of Medicine, Queen Savang Vadhana Memorial Hospital, 209 Jermjormpol Road, Si Racha District, Chon Buri Province 20110, Thailand

**Keywords:** Brucellosis, Glenohumeral joint, Septic arthritis, Total shoulder arthroplasty

## Abstract

**Introduction:**

The main socioeconomic area in Thailand has been the agricultural endemic area, where brucellosis infection, one of the bacterial infectious diseases has been an overlooked diagnose.

**Presentation of case:**

A 50-year-old Thai woman was admitted to the hospital with pain and limited motion in her left shoulder. These symptoms have been prolonging with progressive clinical for two years. The physical examination revealed stiffness in all directions. The glenohumeral joint appeared to be narrowing on radiographic evaluation. The multiple loculate mass and septic glenohumeral joint arthritis were seen by magnetic resonance imaging (MRI). Furthermore, the Brucellosis investigation specificity was Brucella IgM/IgG positive. This patient was treated with a combination of surgery and oral antimicrobial medication. We decided to perform a total shoulder arthroplasty because the patient was still in pain and stiff from secondary arthritis.

**Discussion:**

As a result of the late treatment for osteoarticular involvement, secondary osteoarthritis develops until leading to significant cartilage loss. Therefore, even medical treatment and surgical debridement, the patient still suffers from secondary osteoarthritis, which causes pain and limited activity. The arthroplasty treatment method plays a role in the treatment of function following secondary osteoarthritis infection.

**Conclusion:**

The role of arthroplasty was selected in the treatment of brucellosis osteoarticular involvement, which is an uncommon and difficult to identify condition that can lead to maltreatment. So that this case report offers the treatment option if the patient was not responsible for the medical and surgical debridement therapy with secondary osteoarthritis at the glenohumeral joint, even though the brucellosis infection condition had completely resolved.

## Highlights

• Main socioeconomic area in Thailand has been the agricultural endemic area, where brucellosis infection has been an overlooked diagnose.
• The late treatment for osteoarticular involvement leading to the secondary osteoarthritis development.
• Non-respond the medical and surgical debridement therapy with secondary osteoarthritis at the glenohumeral joint, even though the brucellosis infection condition had completely resolved.
• Arthroplasty treatment plays a role in the treatment of function following secondary osteoarthritis infection.

## 1 Introduction

Brucellosis is characterized by Brucella species, a gram-negative coccobacillus that is oxidase positive, encapsulated, and immobile in domestic animals. The Brucella species, *M. tuberculosis*, *C. burnetii* and endemic fungus are all related to epidemiologic variables. Thailand's main occupation is farmer and agriculture, which is directly related to animal production. This bacterial infection has been recognized as one of the socioeconomic risk factors that influence zoonosis. Consumption of unpasteurized dairy products such as milk, cream, butter, and fresh cheese, as well as occupational contact with animals on a farm in an epidermic area, increased the risk of behavior [1], [2].

The symptoms were non-specific and not clearly identifiable, including numerous organ systems such as the hepatobiliary, bone and joint, and lymphatic systems. The musculoskeletal systems have been described in several case studies, indicating that this type of Brucellosis is most likely to infect these systems. The prevalence of musculoskeletal systems was determined to be anywhere between 10 % and 85 %. Spondylitis, extraarticular soft tissue involvement, tenosynovitis, and bursitis are the most common brucellosis symptoms [3].

In a patient with septic arthritis, the erythrocyte sedimentation rate (ESR) and C-reactive protein (CRP) levels are frequently increased. The white blood cell count and procalcitonin level may be elevated or normal. Blood culture or tissues collected from synovial fluid or bone marrow are the gold standard for diagnosis. The sensitivity of blood cultures varies between 17 and 85 %. In cases of osteoarticular brucellosis, the development time of brucella bacteria in blood culture was much shorter, according to certain investigations [4].

The others profile often dominates in brucella arthritis lymphocytes, whereas polymorphonuclear leukocytes frequently predominate in high-virulent bacterial infections. Bacteria for culturing should be taken from blood that has been properly diagnosed using serological procedures such as standard tube agglutination (STA) and enzyme-linked immunosorbent assay (ELISA). STA (Wright) measures the total amount of immunoglobulin M (IgM)/immunoglobulin G (Ig G) antibodies [5].

In the case of secondary osteoarthritis that has not responded to medical or surgical debridement, the role of arthroplasty in prolonged osteoarticular involvement infection seems to be the only treatment option. Brucellosis infection is uncommon, and it is unclear when and for how long the patient underwent the arthroplasty. Finally, the case study suggests arthroplasty as a therapy option to improve quality of life and guide future decision making in brucellosis osteoarticular involvement.

## 2 Presentation of case

A 50-year-old Thai female patient presented with a two-year history of pain and stiffness in her left shoulder and came to the orthopedic outpatient department. There were not any medical conditions. There has been no traumatic event in the patient's condition. She had a swelling on her left shoulder two years ago, and her pain has reduced her range of motion in all directions over the last six months. Due to these complaints, the patient had previously received NSAID analgesic drugs and was injected with subacromial steroid injection treatment at a nearby hospital. The patient did not respond to these treatments and presented with the same problems to our hospital. There were no other underlying disorders found in the patient's history. No discomfort, fever, weight loss, night sweats, or other joint problems were present. A farmer raising a goat was the occupational patient. She stated that he had had raw dairy products on occasion, notably fresh goat milk. We recorded a patient data following by Surgical Case Report criteria 2020 (SCARE 2020 criteria) [6].

**Fig. 1:**
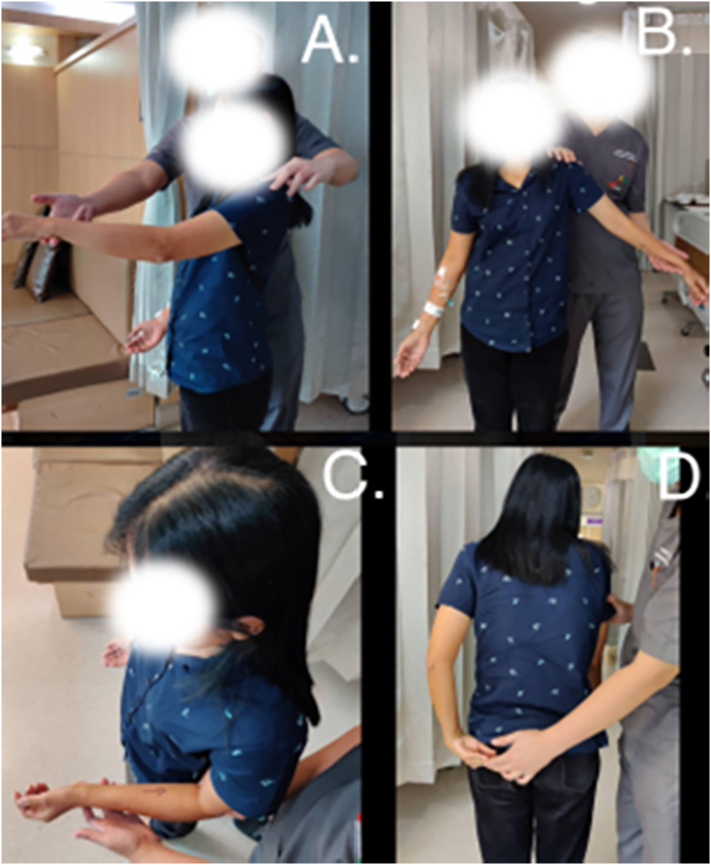
The range of motion of the left shoulder a.) forward elevation motion, b.) Abduction motion, c.) external rotation arm as side motion and d.) internal rotation motion.

Only swelling of the left glenohumeral joint and mobility restriction of the left glenohumeral joint were found on physical examination (Fig. 1). The passive range of motion, the forward elevation was 90 degrees, Abduction 90 degrees, external rotation (arm as sided) was 90 degrees and internal rotation was L4 level. The anterior deltoid manifestation presented an 8 × 3.5 cm anterior deltoid mass which is cystic and movable.

Laboratory findings included the white blood cell count 12,360 /mm3 (normal range: 3500-11,000/mm3), hemoglobin 14 g/dL (normal range: 13.2–17.3 g/dL), platelet count 150,130 /L (normal range: 150,000–450,109 /L), C-reactive protein (CRP) 13.3 mg/L (normal range: 0.1–5 mg/L), and erythrocyte sedimentation rate (ESR) 28 mm/h (normal range: 0–15 mm/h).

**Fig. 2:**
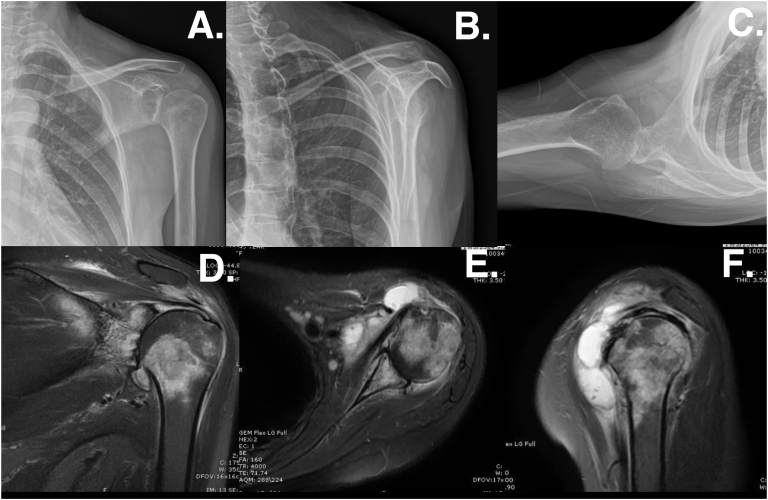
The radiographic imaging of the left shoulder showed that the narrowing and attrition between glenoid and humeral head a.) Anteroposterior view, b.) Transcapular Y view and c.) Transaxillary view. The MRI T2FS of the left shoulder showed d.) Coronal view T2FS, e.) Axial view T2FS and f.) Sagittal view T2FS.

There were positive findings matching the left glenohumeral joint narrowing and sclerosis glenoid side on X-ray (Fig. 2A, B, C). Magnetic resonance imaging of the left shoulder joint revealed subacromial space (subacromial bursa) and glenohumeral joint effusion with high signal intensity in the coronal T2FS images. A loculated effusion was observed in the anterior deltoid and subscapularis upon magnetic resonance imaging in the axial T2FS. The cartilage of the glenohumeral joint was subchondral bone marrow edema, loss of the cartilage layer of the glenohumeral side and intact the rotator cuff tendon in the coronal and axial T2FS (Fig. 2D, E, F).

**Fig. 3:**
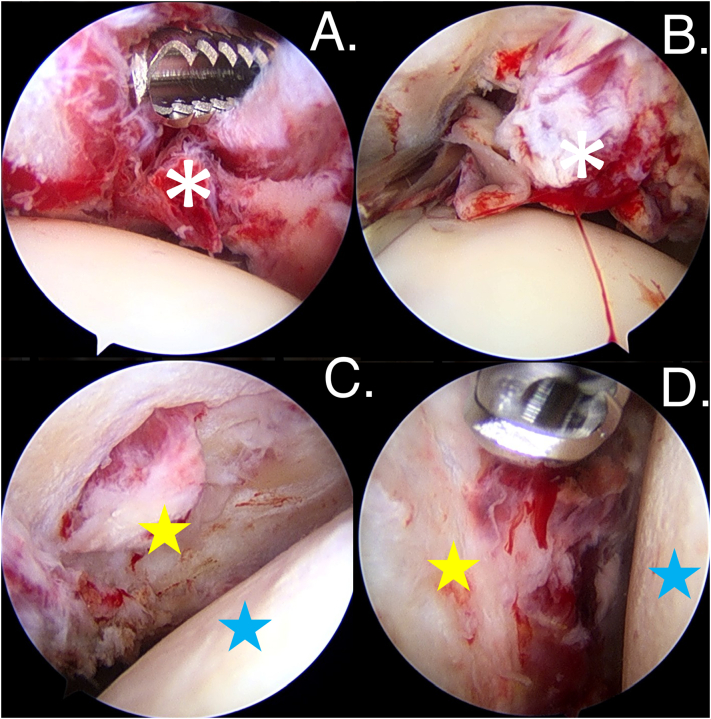
The intraoperative imaging from the arthroscopy from the posterior viewing portal of the left shoulder showed that a., b.) a lot of inflammatory tissue into the glenohumeral joint (white asterisk) and c., d.) the glenohumeral cartilage was loss both humeral head (blue star) and glenoid side (yellow star). (For interpretation of the references to colour in this figure legend, the reader is referred to the web version of this article.)

At the left shoulder, the first arthroscopic procedure was an arthroscopic debridement, synovectomy, and pan capsular release with manipulation. The intraoperative tissue finding was showed a lot of inflammatory tissue and severe hyperemic synovial tissue hyperplasia with fibrinoid necrotic tissue both of intraarticular and subacromial space. Furthermore, the glenohumeral cartilage has been severely damaged, exposing the subchondral bone. The aberrant tissue was taken away to be cultured and histologically examined (Fig. 3).

There was bacterial growth in blood cultures which gram negative bacilli suspected brucella spp. (urease rapid positive). The lab of specificity of the Brucellosis was Brucella IgM/IgG positive (ELISA- IgG > 100 U/ml and IgM > 21.80 U/ml).

**Fig. 4:**
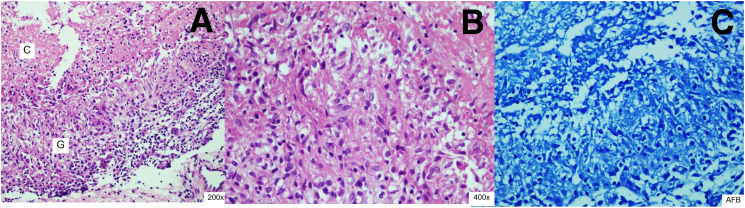
a.) H&E 200×, the picture shows caseating granuloma composed of aggregation of epithelioid cells (G) with center of caseous necrosis (C), b.) H&E 400×, the picture shows epithelioid cell aggregates admixed with small lymphocytes. c.) AFB stain, No Mycobacterium is identified on AFB stain.

The patient underwent operation for histopathological diagnosis. The pathology report, as the result is necrotizing granulomatous inflammation, no Mycobacterium on AFB stain, no demonstrated fungi, and GMS stain and negative for malignancy. Histopathological, the lesion reveals caseating granulomas comprising of central caseous necrosis surrounded by aggregate of epithelioid cells and lymphocytes. Rare Langhans giant cells are present. No microorganisms are demonstrated on AFB stain. The *M. tuberculosis* complex PCR, Mycobacterium (NTM) PCR were negative finding. The fungi culture was no growth for fungus (Fig. 4).

Patient was treated initially with doxycycline (100 mg 2 × 1 per oral) and rifampicin (600 mg 1 × 1 per oral) for four months after the first arthroscopic debridement. The patient's treatment was completed in 16 weeks. At the end of the medical treatment with anti TB drug and oral antibiotics, the follow up sedimentation rate and CRP levels were slightly decreased until the normal range after taking medication for 16 weeks. Because she still had pain and stiffness in her shoulder, the second arthroscopic debridement was performed. We explained with the patient that the purpose of this operation would be to ensure that the infectious condition had subsided and that he was prepared to continue with the arthroplasty procedure. The inflammatory tissue was reduced intraoperatively, causing uncomfortable and stiffness shoulder that resulted from the prolonged infection, eventually leading to secondary osteoarthritis of the glenohumeral joint.

**Fig. 5:**
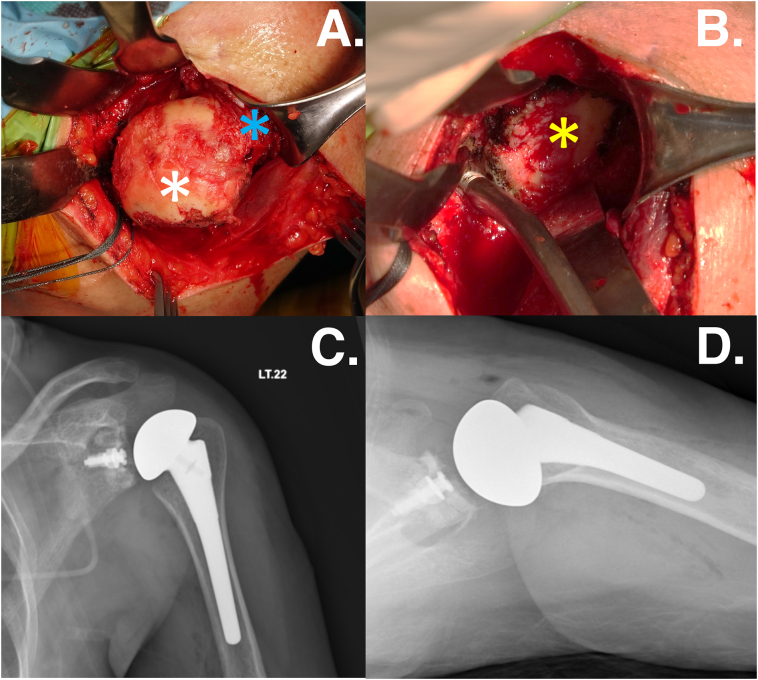
The intraoperative of the left total shoulder arthroplasty showed a.) the severe cartilage loss of the humeral head (white asterisk) and the rotator cuff was intact (blue asterisk). b.) the severe cartilage loss of the glenoid was exposed (yellow asterisk). The postoperative x-ray after performing the cementless total shoulder arthroplasty at the left shoulder. c.) the anteroposterior view and d.) the trans axillary view. (For interpretation of the references to colour in this figure legend, the reader is referred to the web version of this article.)

The tissue diagnosis was evaluated again, the ELISA Brucella IgM/IgG was negative including to the hemoculture was not found the microorganism. The ESR and CRP were within normal limits, with no signs of infection occurring. We decided to do a cementless total shoulder arthroplasty on the patient's left shoulder after 1 month by high experience sport medicine surgeon. The surgical approach was used by deltopectoral approach. The intraoperative findings revealed that the remnant normal cartilage was just a little amount and that most of the cartilage area had vanished, exposing the subchondral bone (Fig. 5). After three months, the patient's left shoulder function was returned without pain at the orthopedic outpatient department. There were no recurrent infection conditions, and normal activities could recover.

## 3 Discussion

In our case, the involving glenohumeral joint region was at the left shoulder. This is the first case reported to manage with the arthroplasty. The arthroplasty was selected in the event of a prolonged infection period, as brucellosis is a rare and difficult to identify disease that can lead to neglect. If the infectious disease is not treated in the inappropriate way. The patient's symptoms will worsen until the clinical and anatomy of the structural damage has progressed to irreversible conditions. This case report would be one of the options for treatment and literature reviews have been reported the several ways to treatment in the shoulder brucellosis infection (Table 1).

**Table 1:** Previous published cases report of the shoulder brucellosis involvement in the literatures (2006–2021).

Author	Year	Patient series	Locations	Treatment
Pourbagher et al. [14]	2006	251 cases with 1 case subacromial bursitis	Subacromial bursitis	Two or three drug combination: ciprofloxacin, tetracycline, rifampicin, streptomycin for 45 days.
Bosilkovski et al. [15]	2016	331 cases with 12 shoulder involvement cases	Shoulder	Two or three drug combination: streptomycin, gentamicin, doxycycline, rifampin, ciprofloxacin, and co-trimoxazole for 45–60 days
Konya et al. [16]	2018	1 case (mimic soft tissue tumor)	Brucellar Granulomatosis shoulder	Surgical management: mass resection Medical management: rifampicin 600 mg po and doxycillin 200 mg po for 12 weeks.
Bilgehan et al. [17]	2019	1 case	Subacromial and subdeltoid bursitis	Doxycycline (100 mg 2 × 1 per oral) and rifampicin (600 mg 1 × 1 per oral) for six weeks.
Fu-Sheng Wang et al. [18]	2021	1 case	Subacromial bursitis	Surgical management: arthroscopic debridement Medical management: rifampicin 750 mg qid combined with doxycycline 100 mg bid for 6 weeks

The prevalence of brucellosis is influenced by several risk variables, including main occupation, native region, preparing meals, and dietary behavior. The major endemic areas include the Mediterranean region, the Middle East, Central Asia, China, India, and Sub-Saharan Africa, where agriculture and farming are the dominant occupations [7]. The brucellosis was transmitted through direct contact when people ate contaminated animal products (e.g., unpasteurized milk, undercooked raw meat, and unpasteurized cheese). According to those studies, human-to-human transmission occurs rarely and is caused by direct contact with contaminated tissues or aerosols through skin lesions or conjunctiva [8].

The hip, knee, shoulder, and ankle were all determined to have brucellosis peripheral arthritis. If the patient does not find the microorganism early, the infection will progress to septic arthritis, with abscess formation as a severe complication. The inflammatory process from the prolonged septic arthritis will slowly erode the cartilage and capsular. As a result, arthroplasty may be beneficial in the treatment of chronic osteoarticular arthritis [9], [10].

The main clinical presentation is an inflammatory sign, particularly joint involvement, which manifests as tenderness surrounding the specific joint with pain when moving the affected joint. Fever, chills, nocturnal sweating, and myalgia are among the non-specific symptoms. The total number of IgM/IgG antibodies will be positive by a serology test from synovial tissue or synovial fluid in brucellosis. Standard agglutination test (SAT) titer ≥1:160 is in favor of brucellosis diagnosis [11].

Nowadays, brucellosis is treated using a combination therapy that includes nonstandard medication for individual brucellosis infections. Doxycycline, streptomycin, gentamicin, ciprofloxacin, trimethoprim/sulfamethoxazole, and rifampicin are the most common antimicrobial used. The course of treatment was extended for at least 3 months to prevent brucellosis relapse. Debridement played an important role in surgery from acute to chronic, and currently, osteoarticular joint involvement is frequently treated with arthroscopic non-invasive surgical debridement. If an abscess develops, surgical intervention will be required to treat it [12]. In the case of a specific group of patients with spinal abscess, vertebral collapse, bone destruction, or cord compression, surgical treatment could be indicated [13].

## 4 Conclusion

This is the first case report of a total shoulder arthroplasty following a brucellosis infection in the glenohumeral joint. Brucellosis infection is uncommon in the general population, except for agricultural domesticated animal farms, which are primarily occupational. This microorganism was shown to cause clinical symptoms, necessitating further investigation, advanced imaging, and the experience of a pathology specialist. For whatever reason, brucellosis was neglected and misdiagnosed until the glenohumeral joint was destroyed. Even though the brucellosis infection has completely resolved, the patient continues to experience pain and stiffness due to secondary osteoarthritis. As a result, arthroplasty will offer as a treatment option with a favorable outcome and a significantly improved quality of life.

## Sources of funding

None.

## Informed consent

Informed consent was obtained from individual participants included in the case report.

## Ethical approval

The Human Research Ethics Committee of Thammasat University Medicine and the study was approved by the ethics committee/IRB of MTU-EC-OT-0-131/65 on 28/06/2022. The Clinical Trial Registration: Thailand Clinical Trials Registry approved by TCTR Committee on 6/06/2022. The TCTR identification number is TCTR20220607001.

## Declaration of competing interest

The authors declare that they have no conflict of interest.
